# Practice modality of motor sequences impacts the neural signature of motor imagery

**DOI:** 10.1038/s41598-020-76214-y

**Published:** 2020-11-05

**Authors:** Britta Krüger, Meike Hettwer, Adam Zabicki, Benjamin de Haas, Jörn Munzert, Karen Zentgraf

**Affiliations:** 1 Institute for Sports Science, Justus Liebig University, Giessen, Germany; 2 Max Planck School of Cognition, Leipzig, Germany; 3 Institute of Sport Sciences, Goethe University Frankfurt, Ginnheimer Landstrasse 39, 60487 Frankfurt am Main, Germany; 4 Experimental Psychology, Justus Liebig University, Giessen, Germany

**Keywords:** Human behaviour, Cerebellum, Motor cortex, Premotor cortex

## Abstract

Motor imagery is conceptualized as an internal simulation that uses motor-related parts of the brain as its substrate. Many studies have investigated this sharing of common neural resources between the two modalities of motor imagery and motor execution. They have shown overlapping but not identical activation patterns that thereby result in a modality-specific neural signature. However, it is not clear how far this neural signature depends on whether the imagined action has previously been practiced physically or only imagined. The present study aims to disentangle whether the neural imprint of an imagined manual pointing sequence within cortical and subcortical motor areas is determined by the nature of this prior practice modality. Each participant practiced two sequences physically, practiced two other sequences mentally, and did a behavioural pre-test without any further practice on a third pair of sequences. After a two-week practice intervention, participants underwent fMRI scans while imagining all six sequences. Behavioural data demonstrated practice-related effects as well as very good compliance with instructions. Functional MRI data confirmed the previously known motor imagery network. Crucially, we found that mental and physical practice left a modality-specific footprint during mental motor imagery. In particular, activation within the right posterior cerebellum was stronger when the imagined sequence had previously been practiced physically. We conclude that cerebellar activity is shaped specifically by the nature of the prior practice modality.

## Introduction

During the last decade, phenomena of embodied cognition have attracted much attention in the field of cognitive neuroscience. The key underlying idea is that cognition is not processed in isolation from the body, but is shaped by bodily states and body-related experiences^1–3^. Within this framework, motor imagery (MI) has been considered to be a body-based simulation process that uses the motor system as a substrate^2,4^. In MI, subjects imagine the execution of a bodily movement from a first-person perspective, emphasizing a strong kinaesthetic component but without overt movement^5^. With regard to the neural substrate, MI is believed to be organized around several core and broader motor regions: the supplementary motor area (SMA), the different sections of the premotor cortex (dPMC, vPMC), the primary motor cortex (M1), posterior parietal regions such as the inferior (IPL) and the superior parietal lobe (SPL), the basal ganglia (BG), and the cerebellum^6–10^. However, the premotor area, the posterior parietal lobe, and the cerebellum seem to be the critical structures for performing MI^11^.

Computational models in motor control suggest that these areas store and process internal forward models that predict sensory consequences given the current state and the motor command^12–15^. Blakemore and Sirigu^16^ suggested that internal models are also used in MI, and that it is particularly the retrieval of the stored forward model, which estimates the anticipated sensory outcome of the movement, that also seems to be used in motor imagery^16–20^. Kilteni et al.^21^ demonstrated impressive support for this notion, showing that motor imagery produces somatosensory attenuation just like executed movement does. This indicates that sensorimotor prediction might be the mechanism that makes MI and motor execution (ME) equivalent.

In applied contexts, not only physical practice but also the additional implementation of mental rehearsal has become an important technique for improving motor performance in athletes and patients^22,23^. Behavioural studies using MI have shown improvements in speed, strength, and accuracy of motor execution^24–27^. Trial-by-trial monitoring in phases of skill learning has further revealed a similar asymptotic learning curve during MI and physical practice^28^. These findings support the notion that MI is not just epiphenomenal, but plays a functional role in the cortical plasticity related to performance. Regarding actual plasticity effects on cortical and subcortical structures, Lacourse et al.^29^, for instance, showed that physical and mental practice of a finger-sequence task leads to similar shifts in activation in several sensorimotor structures. In an early study, Pascual-Leone et al.^30^ used TMS to map M1 during a five-day intervention in which training groups had to either execute or imagine a five-finger piano sequence. Along with progressive performance improvements, they demonstrated that both training groups showed comparable modulations in cortical representations within M1, underpinning the notion of congruent training effects of mental and physical training. Looking at these findings, it can be argued that structural and functional changes might arise from two sources: not only from our actual bodily experience and body state estimation but also from our capacity to imaginatively recreate bodily experiences^31^. However, it is still under debate whether neural plastic changes related to the different training modalities can possibly be the same due to the lack of movement-related sensory feedback in MI. Furthermore, it is not yet clear which impact different training modalities have on the mere simulation of a movement. Two central features of prior studies in the field are that they particularly investigated either the impact on ME and MI of different training modalities on motor execution^29,32^ or the effect of interventions in just one training modality^33–36^.

Against this background, the present experiment examined possible modulations in neural activation patterns induced by mental and physical practice in one group of subjects using a motor imagery paradigm. The aim was to try to disentangle whether mental or physical practice modalities form specific neuronal imprints during a mental simulation process. More precisely, we aimed to elucidate whether MI uses the same neural substrate organized around cortical (PMC, M1, PPC) as well as subcortical (basal ganglia, cerebellum) motor areas irrespective of mental, physical, or no previous practice of movement sequences. We focused predominantly on premotor, posterior parietal, and cerebellar areas that prior work has demonstrated repeatedly to be of mandatory importance for mental simulation processes such as MI^7^. Subjects participated in a two-week practice intervention during which they mentally and physically rehearsed different hand movement sequences. During fMRI scanning, they imagined physically trained, mentally trained, or untrained sequences. We then analysed the collected fMRI data to test whether mental and physical practice of the sequential motor task left an equivalent versus modality-specific footprint in our regions of interest (ROIs). In a first step, we defined neural activation sites of MI irrespective of prior experience. In a second step, we compared MI of the differentially trained sequences by comparing MI of the physically trained with MI of the mentally trained sequences in order to describe possible quantitative activation differences related to different forms of prior practice modality. Based on the notion that forward modelling is processed in MI and ME, but that a crucial need to integrate the output of the forward model with actual sensory feedback is found only in ME, we hypothesized that physically trained sequences would lead to a more pronounced neural imprint within motor and motor-related areas such as premotor, posterior parietal, and cerebellar cortices.

## Results

### Behavioural data

**Figure 1:**
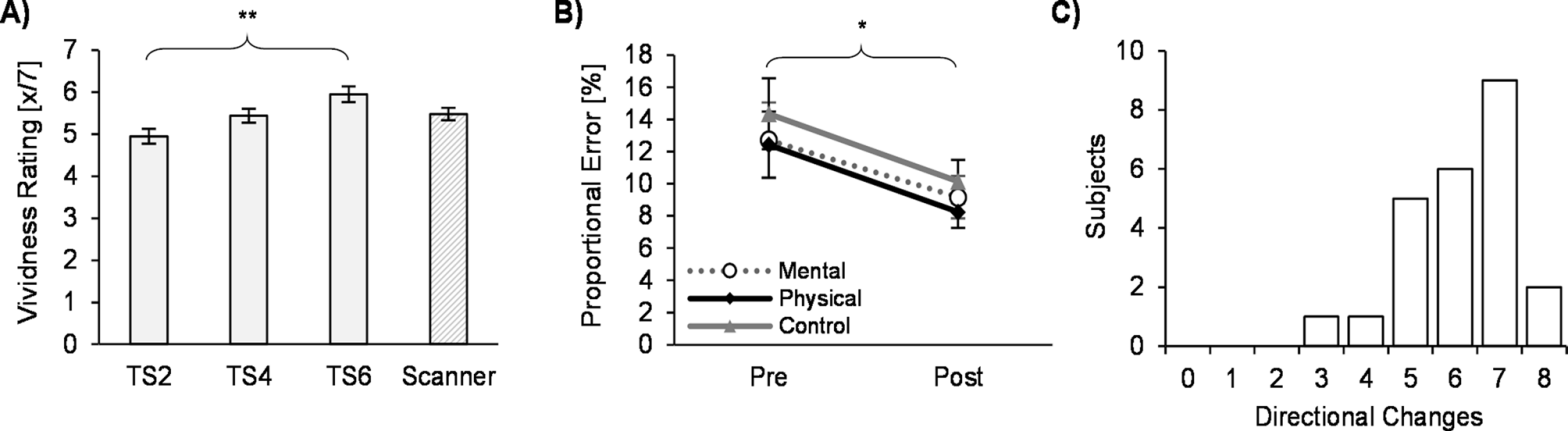
Behavioural Results. (**A**) Vividness of MI ratings as indicated on a scale from 1 (lowest) to 7 (*highest*), after the 2nd (TS2), 4th (TS4), and 6th (TS6) training session as well as after the scanner session. (**B**) Proportional error of sequence execution as compared to the presented model pre- and post-practice interventions. (**C**) Distribution of the number of directional changes in eye movement measured using electrooculography. Error bars indicate standard error of the mean (SEM), *p < .05, **p < .01.

Vividness ratings for imagery of hand movement sequences trained during practice sessions increased progressively throughout the intervention as assessed with a 7-point scale ranging from 1 (*very low*) to 7 (*very high*) (*M*_TS2_ = 4.95 ± 0.88; *M*_TS4 _= 5.44 ± 0.82; *M*_TS6_ = 5.95 ± 0.71; *F*(1.3, 21.7) = 10.10, *p* = 0.002, η_p_^2^ = 0.39, Greenhouse–Geisser-corrected) (Fig. 1A).

During practice, subjects’ execution speed and spatiotemporal accuracy improved in all conditions (Fig. 1B). A two-way repeated measures ANOVA revealed a significant main effect of time, showing significantly decreased proportional errors in temporal accuracy across the training intervention, *F*(1, 23) = 7.67, *p* = 0.011, η_p_^2^ = 0.25. There was a positive correlation between execution and imagery durations, *r* = 0.47, *p* < 0.001, which was strong for physically trained sequences, *r*_*Physical*_ = 0.70, *p* < 0.001, and moderate for mentally trained sequences, *r*_*Mental*_ = 0.52, *p* = 0.010, as well as for sequences that were not trained at all, *r*_*Control*_ = 0.44, *p* = 0.032. During the scanner session, there was no systematic difference in imagery durations between the three conditions (*M*_Mental_ = 4.29 ± 0.79 s, *M*_Physical_ = 4.32 ± 0.81 s, *M*_Control_ = 4.28 ± 0.77 s; *F*[2, 69] = 0.01, *p* = 0.99, η_p_^2^ = 0.00).

Analysis of EOG data showed an average of 6.13 ± 1.22 turning points in task-related eye movements of 24 subjects (Fig. 1C). Considering that each sequence consisted of six targets, compliance with task instructions was represented by six sequence-related eye fixations at turning points as well as an additional shift when eyes moved back to the starting position. Additional behavioural results are reported in the supplementary materials.

### Neuroimaging data

In a first step, we identified brain regions associated with motor imagery of sequential finger movements irrespective of prior experience by contrasting all imagery conditions against rest (Table S3) and performing a conjunction analysis over all imagery conditions. In a second step, we identified brain regions involved in the differential processing of MI depending on prior experience.

#### Imagery of movement sequences

**Figure 2:**
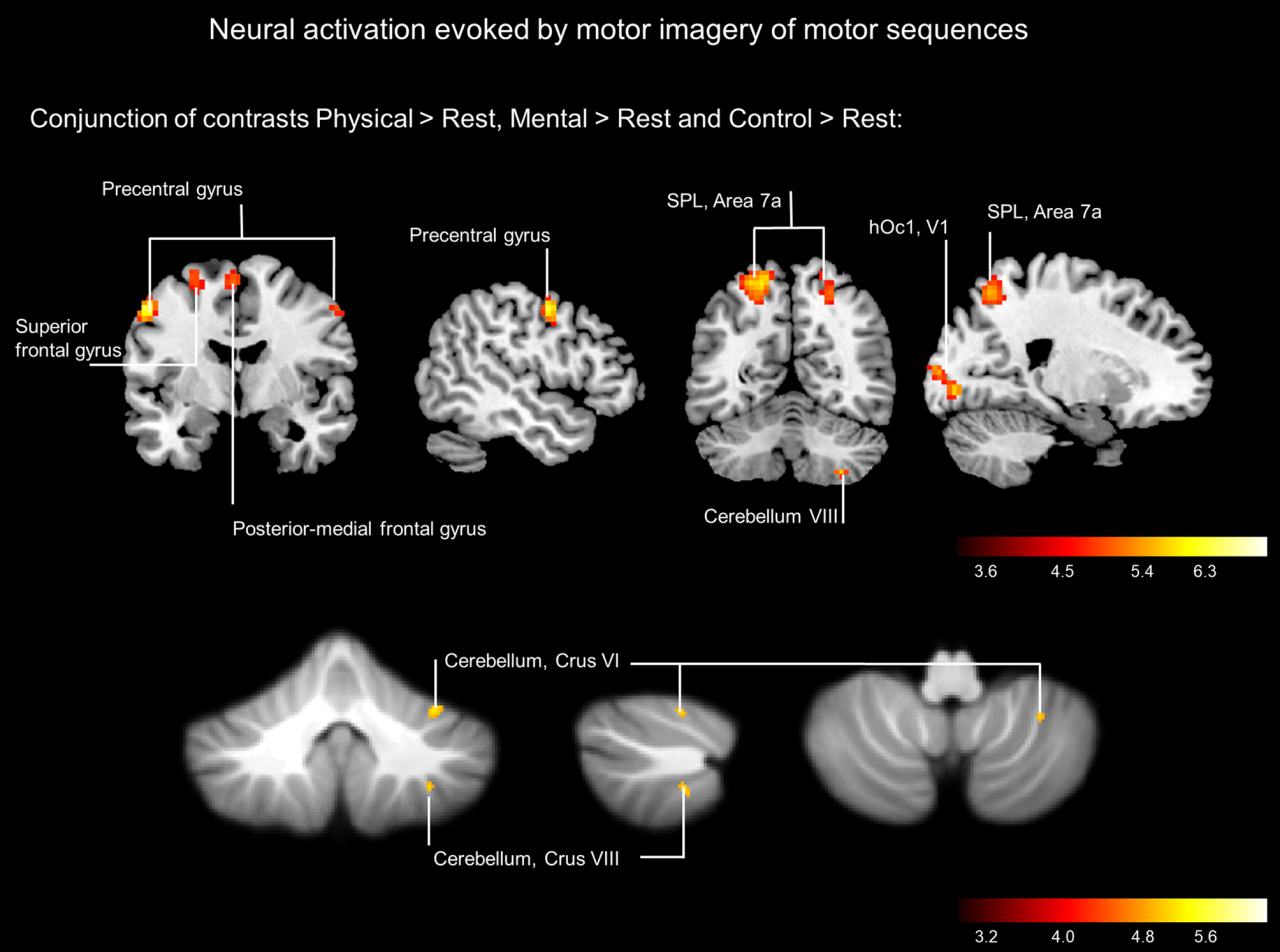
Neuroimaging Results. Activation during MI of the six hand movement sequences revealed by conjunction analysis of physically trained, mentally trained, control sequences (*p* < 0.05, FWE-corrected).

The conjunction analysis of all experimental conditions (physical, mental, control) compared to rest revealed significant activation increases in regions previously shown to be involved in motor imagery: motor, parietal, and visual areas as well as in the cerebellum (Fig. 2). More specifically, activation was identified in the SPL (Area 7A), the superior occipital gyrus, the precentral gyrus, the superior and posterior-medial frontal gyrus of the left hemisphere, as well as the right cerebellar lobule VI and VIII (FWE-corrected < 0.05, Table 1). These results were highly consistent with a broad body of literature demonstrating the role of motor and motor-related areas during MI^7^.

**Table 1:** Brain regions identified by conjunction analysis over all imagery conditions (*p* < .05, FWE-corrected).

	Left/Right	Coordinates of max. t value			t value	Suit
*Conjunction Physical ∩ Mental ∩ Control*						
Superior parietal gyrus (Area 7A)	L	− 18	− 64	62	7.70	
Superior occipital gyrus (hOc1, V1)	L	− 18	− 91	2	7.02	
Precentral gyrus	L	− 54	− 4	47	6.82	
Precentral gyrus	R	57	− 1	44	6.13	
Lingual gyrus (hOc1, V1)	R	21	− 85	− 4	6.21	
Posterior-medial frontal gyrus	L	− 3	− 1	62	6.10	
Superior parietal gyrus (Area 7A)	R	21	− 64	56	5.98	
Superior frontal gyrus	L	− 27	− 4	65	5.32	
Cerebellum VI	R	34	− 51	− 24	5.16	x
Cerebellum VIII	R	32	− 50	− 50	5.08	x

MNI coordinates. Cluster size > 20. FWE-corrected *p* < .05.

#### Practice-modality-dependent activation during MI

**Figure 3:**
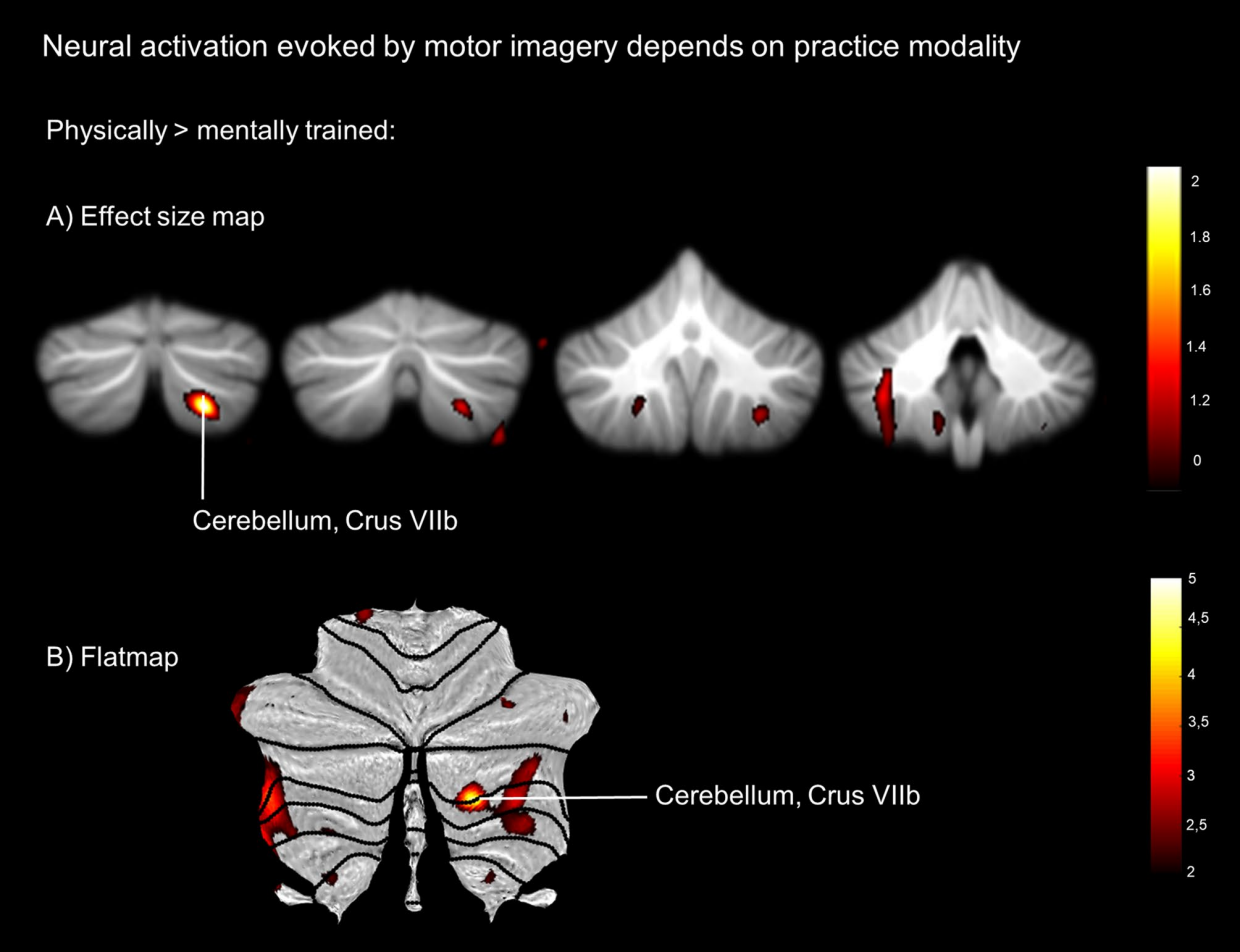
Neuroimaging Results. Significantly stronger activation in physically compared to mentally trained sequences (*p* < 0.05, FWE-corrected). (**A**) Effect size maps using Cohen’s *d* for the contrast of Physical > Mental. Effect sizes were thresholded at *d* = 1.0. (**B**) Flatmap depicting a flat representation of the human cerebellar cortex with a projection of the T map of the contrast Physical > Mental. T map was thresholded at *t* = 2.5.

A ROI analysis involving those areas generally activated during MI revealed an experience-dependent activation increase for ME compared to MI practiced sequences within the cerebellum of the right hemisphere (Crus VIIb, x = 22, y = − 76, z = − 49; FWE-corrected < 0.05) (*Fig. **3*, Table 2). We further performed an exploratory analysis using a less rigorous threshold. The respective results are reported in the supplementary materials.

**Table 2:** Brain areas identified by contrasting imagined motor sequences that were physically or mentally practiced (*p* < .05, FWE-corrected ROI analysis).

	Left/Right	Cluster size	Coordinates of max. t value			t value		Suit
*Physical vs. Mental*								
Cerebellum Crus VIIb	L	21	− 32	− 44	− 42	3.80	x	
Cerebellum Crus VIIb	R	80	22	− 76	− 48	4.93*	x	

MNI coordinates. *p < .05, FWE-corrected ROI analysis. *p* < .0001, uncorrected. Mask size = 13,236 voxels (1 × 1x1 mm) including both hemispheres.

## Discussion

We tested whether the neural imprint within the cortical as well as subcortical motor areas of an imagined manual pointing movement sequence is determined by the nature of prior practice modality. We found that mental and physical practice of the sequential motor task leaves a modality-specific footprint during mental motor imagery, especially within the posterior cerebellum. It has been suggested that the latter is involved in sensorimotor processing, abstract functions of motor actions, including the implementation of forward models^37–42^, and motor inhibition while performing MI^22^. Generally, the present results indicate that the generation of predictions as well as the availability of sensory feedback, which could be integrated during practice, drives a modality-specific impact on cerebellar activation during MI. That is to say, our data reveal that neural activity within posterior cerebellar regions distinguishes between imagery of mentally trained versus physically trained sequences, whereas both training modalities lead to similar performance increases regarding the spatiotemporal accuracy of imagined sequences.

Based on the present results, we conclude that cerebellar activity is shaped specifically by the nature of the practice modality. Prior motor experience leads to more pronounced activation particularly within the cerebellum. This suggests that practice leads to the development of modality-specific neural imprints even when participants are asked to only simulate trained movements. In this regard, we would argue that functional changes arising from actual bodily experience versus our capacity to imagine bodily experiences have specific neural substrates. This substrate is then used to process an offline motor state that is a prediction based on representations of prior experiences.

On the behavioural level, our data showed an increased performance reflected by spatiotemporal accuracy for physically and mentally trained as well as untrained sequences. These data revealed that mental as well as physical training relate to a similar performance increase for all simulated motor sequences. Furthermore, they suggest a transfer effect to untrained control sequences. Increasing vividness of motor imagery ratings during the intervention period as well as mental chronometry data from the scanner sessions revealed compliance with the task at all times. Other studies corroborate that imagery ability is also related to the neural MI footprint^43–45^ as well as to motor expertise^6^. To the best of our knowledge, a clear experimental disentanglement of these factors has not been achieved before. The inspection of eye movements via EOG data during MI confirmed that participants engaged in similar imagery behaviour during practice and, assumingly, scanner sessions. This did not just support comparable neural activation but has also been shown to enhance the efficiency of MI training^46^. Because the sample in this study showed high general imagery ability, high vividness of imagery ratings during the intervention, as well as behavioural improvements, it can be concluded that MI training sessions of motor hand sequences were as efficient as physical practice—at least in the present setting.

MI comprises the conscious ability to simulate a movement in one’s imagination. Of necessity, this requires an internal representation of that imagined movement^47^. Based on Jeannerod’s mental simulation theory^48^, multiple studies have investigated a possible functional equivalence between motor imagery and actual execution. Results have demonstrated overlapping activation within several cortical and subcortical motor areas^7^. The congruence of functional neuroanatomy between executed and imagined actions both before and after training has been found repeatedly in PMC and SMA, with less consistent findings in M1, S1, visual cortex, cerebellum, and orbitofrontal cortex^29,36,49–51^. For instance, Nyberg et al.^32^ reported more prominent activation in the cerebellum and SMA following physical practice of a similar finger tapping task, whereas MI practice was associated more with SMA and the visual association cortex. However, one has to consider the different lengths of training interventions in these studies (ranging from an intermediate practice within one scanner session^35^ to extensive 6-week interventions^51^), differences in the choice of effectors, or inconsistent imagery instructions. Furthermore, most implemented a between-subject design leading to the possibility of inhomogeneity across the investigated groups.

In the present experiment, a conjunction analysis of MI of either physically or mentally trained, as well as untrained sequences revealed neural activation clusters in bilateral SPL (Area 7A), precentral gyrus, superior occipital gyrus, the left posterior-medial and superior frontal gyrus, as well as the right cerebellar lobules VI and VIIIa. These areas have previously been associated with MI, sensorimotor control, and specifically with the execution of aiming movements^11,52–55^. Especially, the SPL, as a part of the posterior parietal cortex, is associated with state estimation, sensorimotor integration, movement intention, and decision making^56–60^. Cerebellar activation in lobule VIII has been associated with the sensorimotor processing of, in particular, arm and hand movements^61–63^. In this regard, sensorimotor tasks activated the anterior lobe (lobule V) and the adjacent lobule VI with additional foci in lobule VIII. Motor activation was in VIIIa/b; somatosensory activation was confined to VIIIb^64^. Our analyses also revealed strong activation sites in the visual cortex, which, according to Guillot et al.^65^, is associated particularly with visual rather than kinaesthetic imagery processes. This effect could indeed be linked to the nature of the task, assuming that participants generated an image of the target grid as well as their moving hand during motor imagery and/or eye movements during imagery. In this regard, Krüger et al.^66^ recently investigated task-dependent imagery modality in a large group of participants. Their data showed that for a large set of imagined actions, the sensory impression of a motor image could be explained by the environmental demands of the action. For example, in the case of aiming movements that require a certain degree of precision by hitting a target, visual information might be a key feature of the respective action representation. The consequence is that the prediction and the imagination of this action type might be more related to visual aspects of the action.

It is well documented that the cerebellum is one of the critical structures for performing MI^67–70^. Indeed, it has been demonstrated that increasing activation in anterior cerebellar areas is associated with spatial accuracy demands of imagined pointing movements^71^. The cerebellum is known to store internal models and action representations built up by individual motor learning processes (that were modulated here by experiencing different sequences either purely mentally or physically)^20,72^. More specifically, it has been discussed as a principal brain area for the storage of internal forward models that predict movement outcomes supporting predictive motor control^14,73–77^. Thereby, the cerebellum in particular seems to play an important role by providing precise timing information for predictions^78^. Alongside its role in sensorimotor control, several findings provide support for a broader concept of cerebellar function by highlighting the involvement of the cerebellum in diverse cognitive processes such as attention and working memory^79^. Based on the present data, it can be reasoned that cerebellar activity during MI is tuned specifically by physical training (see also Nyberg et al.^32^ for an ME task). Physical practice seems to build up a modality-specific footprint in the cerebellum that is then used to perform an offline mental simulation of the respective movement. Interestingly, several studies have observed that motor expertise is associated with an increase of cerebellar activation during action simulation (observation and anticipation tasks), suggesting that it is particularly motor-related cognitive functions of the cerebellum that can be modified by physical experience^80–82^.

When characterizing the different experiences in the present experiment, it can be stated that a simulation of a movement in the imagination comprises a very conscious and effortful act that requires the retrieval of a stored forward model of that particular movement in every single trial during training. This mental simulation runs offline and is therefore not associated with physical execution. This means that sensory feedback is totally lacking, and, therefore, might not be represented in a significant manner during practice. During physical practice, however, participants are receiving both visual and sensory feedback on the performed movement. Thus, it is evident that both practice modalities are not totally equivalent when it comes to incoming information and feedback on the planned movement. In this context, Zabicki et al.^11^ have argued that execution and imagery representations are neither purely distinct nor purely equivalent. They are best captured by models assuming that ME and MI are distinguishable from each other while preserving a low-to-moderate degree of similarity; and it appears that the similarity between MI and ME is highest on this level of action plans. Heuer^83^ already discussed the idea that different movement characteristics will be learned when real sensory feedback is lacking. Therefore, in the present case, mental practice might build up a rather spatial representation of the given task, whereas physical training also generates a kinaesthetic, more embodied representation reflected by the neural imprint in the cerebellum. This specific representation, however, is used for the upcoming offline simulation.

Another line of research has postulated that the posterior cerebellum might be strongly involved in the inhibition of the motor command during motor imagery^9,22,84^. This inhibitory mechanism is thought to prevent efferent impulses triggered through MI from reaching the medullar and muscle levels^85^. Therefore, we might also speculate that it is especially MI of physically trained sequences that requires such an increased inhibition of the motor command^9,22,84,85^.

On a phenomenological level, the specific representation might indeed result in different ways to imagine a movement (conceptualized as an experience-based prediction). Thus, imagers might create motor images based on their individual (motor) experience that affects the way they create the imagined action. Therefore, we suggest that the (motor) imaging human brain seems to access acquired (motor) experiences. The integrated multisensory experience of visual, spatial, and kinaesthetic action aspects is conditional on the upcoming motor images, because individuals ‘re-experience’ the action using their own experience-dependent representations.

One potential limitation of the present results is due to the nature of employing a within-subjects design in which all participants physically rehearsed several sequences, imagined another set of sequences, and had no practice with a third set of sequences. One possible criticism of using such a design to study differential learning effects is that MI and ME training does not occur in a purely MI or ME context. In other words, the different training modalities are not strictly compartmentalized, and training of sequences in one modality could influence the representation of similar sequences trained in the other. However, if this was the case, it would diminish differential effects of the practice modality, thereby making the substantial effect sizes we observed a conservative estimate. At the same time, the statistical power of a within design is superior, because it controls for confounding inter-individual differences. Furthermore, our design did not allow us to probe the effects of practice modality on motor representations during execution. Future experiments could include such a condition in the scanner to test this issue. A more detailed investigation of subjective imagery quality would also facilitate the testing of possible differential effects of practice modality on different sensory qualities of the motor images. Another interesting extension of the present design might be a pre–post approach, because this would offer the opportunity to qualify representational changes over time. Our main hypothesis, however, was that different training modalities (i.e. either a mental or a physical practice intervention) would lead to a distinguishable neural imprint during mental simulation of trained movements. In this regard, the present design is a very conservative approach to test the building up of specific representations by investigating a simulation that is considered to run on these representations.

Our findings imply that imagery-evoked neural activity in the posterior cerebellum depends on the practice modality. Practice-induced changes in the posterior cerebellum form a specific neural substrate that is used to process an offline motor state. Thus, even when you are only thinking of a movement, the involvement of your cerebellum will depend on whether you have practiced this movement mentally or physically. This has implications for practical applications of motor imagery. It will evoke a different representation, which is probably closer to a true simulation, if it is preceded by physical practice as opposed to imagery practice in isolation.

## Material and methods

### Subjects

Twenty-four volunteers (16 female, 8 male, *M*_age_ = 22.2 ± 2.6 years) participated in the present study. All were right-handed (EHI = 92.4 ± 9.9) with normal or corrected-to-normal vision. They reported no history of neurological or psychiatric disorders and no history or current use of any psychoactive medication. Subjects showed good initial imagery abilities for visual (*external*_pre_ = 2.22 ± 0.73; *internal*_pre_ = 1.83 ± 0.53) as well as kinaesthetic (*M*_pre_ = 2.20 ± 0.68) perspectives as revealed by the Vividness of Motor Imagery Questionnaire (VMIQ-2^5^), in which lower scores reflect more vivid imagery experience. For further information on the investigated subjects, see supplementary materials. The study was approved by the local Ethics Committee of the Psychology and Sports Science Department of the University of Münster, Germany. All subjects gave written informed consent prior to the experiment in accordance with the Declaration of Helsinki.

### Design and task

**Figure 4:**
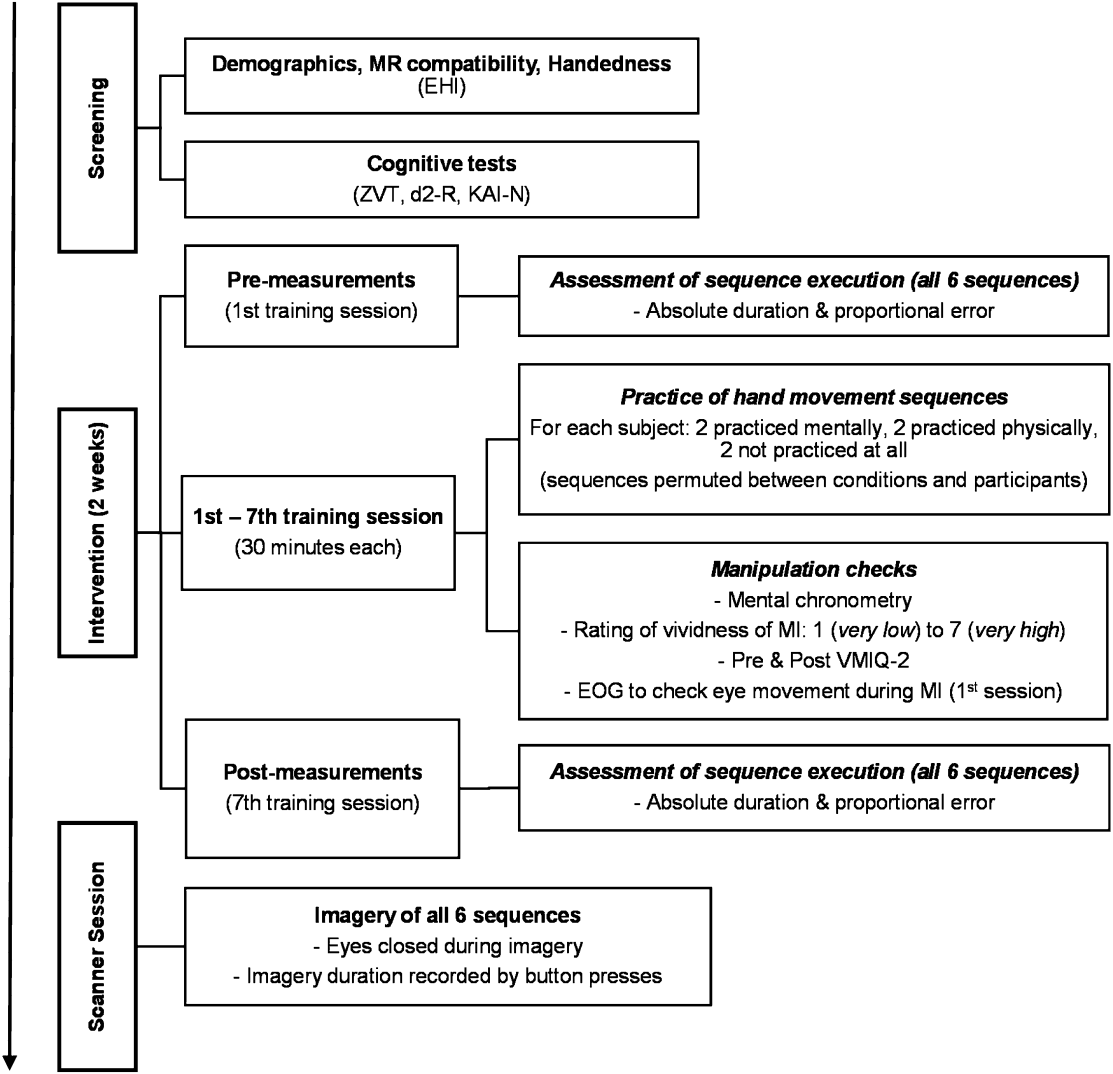
Workflow diagram. Following an initial screening, subjects participated in a two-week training intervention. Throughout the intervention, each subject engaged in mental and physical practice of different hand movement sequences. During the scanner session, subjects imagined mentally trained, physically trained, and untrained control sequences.

The experiment consisted of a two-week training intervention followed by an fMRI scan (Fig. 4). Subjects participated in seven training sessions, each lasting 30–40 min and scheduled for approximately every second day. In each of the seven training sessions, participants trained two sequences mentally and two sequences physically (for a detailed description of the training session, see supplementary materials).

**Figure 5:**
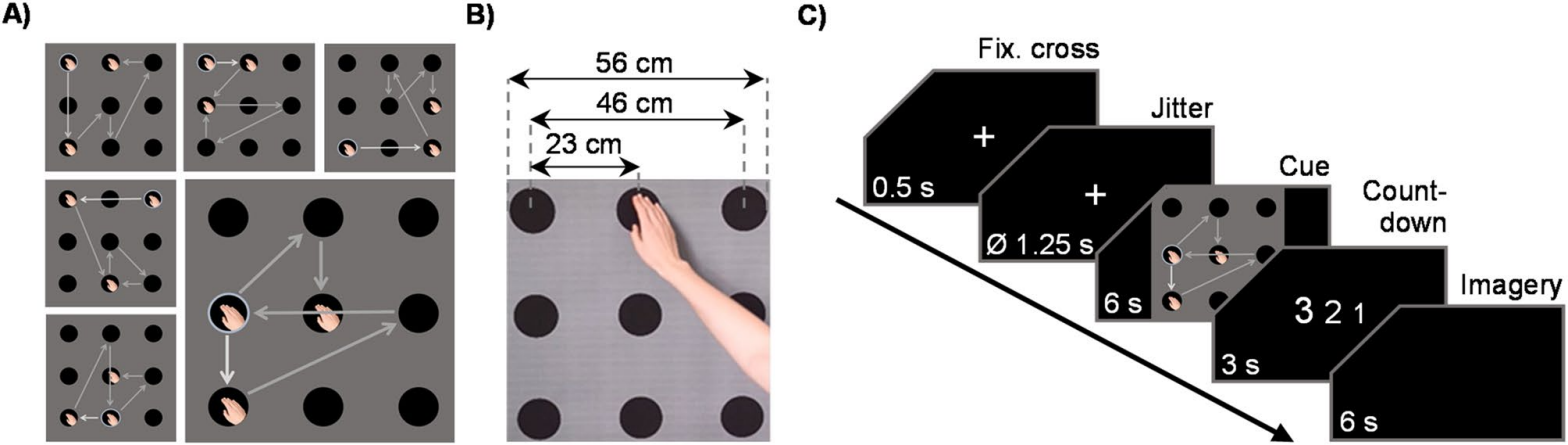
Stimulus material. (**A**) Static arrow images presented in the last block of each practice session as well as during the scanner session and (**B**) videos implemented in practice sessions only. Overall size of the original target grid: 56 × 56 cm, size of targets: Ø 10 cm. (**C**) Temporal structure of the experiment in the scanner.

The study included three mutually exclusive conditions: an MI condition in which sequences were practiced solely mentally without overt movements; an ME condition in which sequences were practiced physically on a target grid; and a control condition in which sequences were not practiced at all. Overall, six movement sequences were designed as aiming tasks in which the right hand moved across the target grid to touch six targets in a given order. Following a within design, all subjects were assigned two sequences for every condition. These sequences were permuted (in pairs) between participants and conditions. For the physical execution, a quadratic 55.5 × 55.5 cm target grid consisting of nine evenly arranged targets (Ø 9.7 cm; see Fig. 5B) was fixed to the wall at participants’ eye level.

During the scanner session, subjects imagined all six sequences regardless of which modality each sequence had been trained in beforehand. For the course of the intervention as well as the scanner session, they were instructed to keep their eyes closed while imagining the hand movements from a first-person perspective. This called for visual and kinaesthetic impressions similar to those present during actual execution as well as emphasizing naturally occurring eye movements.

### Stimuli

The stimulus material consisted of six 6-s video clips showing hand movement sequences and six corresponding static arrow images—that is, one video and one static image were created for each sequence (Fig. 5A + B). All videos were filmed from a first-person perspective. Six targets were touched in each sequence with four adjacent targets and two targets that were further apart. The sequences were matched in terms of displacement path length, number of directional changes, and angular sum. The shown hand always started and returned to the same local position below the target grid and touched one of the outer dots to start a sequence. A PC running Presentation software (v19.0, Neurobehavioral Systems, Albany, NY) was used during both training and the scanner session to present instructions and stimuli. Task progression was self-paced during the training sessions but pre-set for fMRI scanning. During the scanner session, only static arrow images were presented. Instructions and stimuli were projected onto a screen arranged outside the scanner that could be seen through an adjustable mirror attached to the head coil.

### Scanner experiment

Participants performed eleven blocks of twelve trials each (corresponding to two trials in each of the six sequences), amounting to 132 trials and a 50-min scanner session overall. A 6-s rest condition was added that appeared twice every block. Its end was indicated by a sound signal. Sequences from all conditions were presented in a pseudo-randomized order counterbalanced across participants. Each block was introduced by a written instruction presented for 2.5 s (e.g., ‘Please imagine the following hand movement’) followed by a jitter (0–90% of TR) implemented by varying durations of presentation of a fixation cross (Fig. 5C). Static images of the sequences (6 s) or an instruction to rest (2.8 s) were presented followed by the respective imagery or rest phase (6 s). Participants closed their eyes in both conditions and reopened them only to receive instructions. Imagery duration was logged because subjects indicated the beginning and end of their imagery process by button presses when mentally touching the first and last target, again providing a manipulation check as well as information on possible systematic differences in duration of task engagement between training conditions.

### Manipulation checks

In order to monitor subjects’ participation and progress throughout the training sessions, the following manipulation checks were implemented: Prior to the experiment and post-experiment, general motor imagery abilities were assessed using the VMIQ-2^5^. Here, subjects visually or kinaesthetically imagined a set of movements and indicated the perceived vividness on a 5-point Likert scale ranging from 1 (*perfectly clear and as vivid as normal vision*) to 5 (*no image at all*).

After the first and last (7th) training session, subjects physically performed all six sequences so that the sound of hand–wall contacts could be recorded. This allowed accurate temporal identification of absolute duration as well as relative duration of the intervals within the performed sequence. Comparing these to the model provided in the videos revealed progress of temporal accuracy—that is, whether participants showed velocity or temporal proportion changes throughout the training sessions. In addition, possible transfer effects to non-trained sequences were evaluated. In order to assess the degree of temporal congruence, durations of imagery processes during the scanner session and duration of sequence execution as recorded in the post-behavioural session were matched for every individual, providing information on the quality of the temporal organization of the imagined hand movement as well as compliance with the instructions.

Because motor imagery has been shown to be accompanied by eye movements corresponding to the spatiotemporal evolution of imagined movements, electrooculography (EOG) data were acquired once during the first training session^86^. This made it possible to assess and record spatiotemporal patterns of eye movements objectively, thereby controlling active participation and compliance with task instructions and sequence specifics during initial imagery trials. This procedure further allowed us to give subjects feedback on whether they moved their eyes effectively; and it identified participants who did not do so automatically. In the latter case, they received additional instructions to make sure all participants would engage in comparable behaviour during the scanner session. Participants were also asked to rate perceived vividness of their imagery on a 7-point scale ranging from 1 (*very low*) to 7 (*very high*) after every second training session as well as after the scanner session.

### Image acquisition

fMRI data were collected on a Siemens Prisma 3-T whole-body scanner using a 20-channel head coil. A structural image was acquired from each participant consisting of 176 T1-weighted sagittal images (1-mm slice thickness; MPRAGE) and a field map (40 slices; TR = 1000 ms; short TE = 10 ms, long TE = 12.46 ms) using a double-echo gradient echo field map sequence. For functional imaging, 11 runs with 90 volumes per run (i.e. a total of 990 volumes) were registered using a T2*-weighted gradient echo-planar imaging sequence covering the whole brain with 40 slices (slice thickness = 3 mm; 0.75 mm gap; descending interleaved; TR = 2500 ms; TE = 30 ms; flip angle = 87 degrees; field of view = 210 mm × 210 mm). The orientation of the axial slices was parallel to the AC–PC line. Trial onsets were jittered within 0–90% of the TR Image.

### Statistical analysis of behavioural data

Behavioural data on the temporal accuracy of execution were analysed using a two-way repeated-measures ANOVA with time (pre-training and post-training) and condition (mentally trained, physically trained, and control) as within-subject factors. In addition, mental chronometry was assessed by running three separate linear regression analyses between imagery durations in the scanner as indicated by the push of a button and execution times of the post-behavioural session for the three experimental conditions. All behavioural data were analysed using SPSS Statistics (v. 24, IBM inc., Chicago, USA) and MATLAB (v. 2018a, MathWorks inc., Natick, MA). A Shapiro–Wilk test of normality preceded statistical analyses and Mauchly’s test of sphericity was implemented for ANOVAs. Additionally, the number of eye movements during imagery (i.e. EOG data) was monitored in BrainVision Analyzer (v. 2, Brain Products GmbH, Gilching, Germany). Given that participants were instructed to move their eyes corresponding to the imagined movement sequence, episodes of the imagined hand touching a target were indicated by short eye fixations at turning points. The number of these was compared to the model. Ideally, six fixations at turning points could be distinguished as part of the sequence. An additional directional change was identified when eyes moved back to the starting position.

### fMRI analysis

#### Pre-processing

Before conducting statistical tests on fMRI data, spatial and temporal corrections were made in the course of pre-processing using SPM12. To begin with, all collected volumes were realigned to compensate for head movement in the scanner. Field maps were used to generate voxel displacement maps that were then used to unwarp distortions created by differences in susceptibility to the magnetic field. Furthermore, slice time correction was applied to control for slight differences in acquisition times. Functional data were then normalized to MNI space (Montreal Neurological Institute) and co-registered with individual structural images of the participants. As a last step, functional data were smoothed using a three-dimensional Gaussian kernel (9 mm full-width half-maximum [FWHM]).

For the cerebellar data, a specific normalization method was applied to allow a more accurate localization of activation within the small structures of the cerebellum. Because of the low contrast in the 152 ICBM template (MNI space) of the cerebellum, a whole brain normalization that is used as a standard in SPM12 would have led to a large spatial variance between participants^87–90^. Therefore, we used the template of the SUIT toolbox for SPM12 (Version 3.4) that is based on the average cerebellar anatomy of 20 participants. This procedure preserved the fine details of the cerebellum and improved the inter-subject alignment compared to the standard normalization^87^. In a first step, the automatic isolation algorithm provided by the toolbox was used to segregate the cerebellum and the brainstem. If necessary, the isolation maps were corrected manually based on anatomical information, and then these were normalized to the SUIT template via a nonlinear transformation. The resultant deformation maps were used to normalize the functional images of each participant. In contrast to the whole brain data, in which normalization and the ensuing smoothing were performed before the first‐level analysis, in the SUIT normalization, these steps were conducted after the functional data had been analysed on the single-subject level. On the second level, the whole brain and the cerebellar data were analysed in exactly the same way.

#### General linear model and contrasts

First, the six different imagined sequences were matched to the experimental conditions (i.e. mentally trained, physically trained, or control) based on the permutation protocol. Then, first-level analysis was computed using separate general linear models (GLMs) for each subject. Each GLM consists of all eleven runs. For each run, seven boxcar regressors were created corresponding to the six sequences (duration was adjusted to the individual button presses in the scanner) and rest conditions. In addition, the stimulus presentation phase and button presses (with an approximated duration of 100 ms) were included as regressors of no interest. Further regressors were obtained from an in-house *Fmri Artefact Correction Tool* that identifies outlier volumes due to motion during the scan of a volume. Hence, the detection of outlier volumes was based on a comparison of each volume with its two neighbours in a motion‐corrected time series. This procedure was performed by calculating the mean-squared differences to the previous and the next volume. The smaller difference was used as the outlier score for each volume. Scores were thresholded using Hubert and van der Veeken's^91^ method of calculating a skewness‐corrected interquartile range. To threshold outlier scores, the range was multiplied by 1.5 and added to the 75th percentile.

Each regressor was convoluted with a canonical hemodynamic response function. Moreover, six movement parameters from the rigid-body transformation of the motion-correction procedure were entered as covariates. The voxel-based time series were filtered with a high-pass filter (time constant = 128 s).

Then, several contrasts were computed. Practice conditions were contrasted to rest (mental > rest, physical > rest, and control > rest, imagery [including all training conditions] > rest) as well as compared to each other (mental <  > physical, mental <  > control, and physical <  > control). These contrast images were fed into a second-level analysis in order to conduct random effects, group-average analysis. Furthermore, a conjunction analysis identified voxels that were active in all three conditions (mental > rest ∩ physical > rest ∩ control > rest). Significance was assessed via whole-brain analysis (*p* < 0.05, FWE-corrected) and anatomical locations were identified based on MNI coordinates using the Anatomy Toolbox (Version 2.2b^92^) for SPM12. This toolbox was used to label all activations on the basis of cytoarchitectonic probability maps. Significant results within the cerebellum were assigned to the cerebellar lobuli by means of the probabilistic atlas included in the Anatomy toolbox^88^.

For all contrasts comparing the different training conditions against each other, a small-volume correction was conducted with a priori search volumes. These ROIs were selected on the basis of previous findings reported in the literature^7^ as well activation sites observed for the conjunction of all imagery contrasts against rest (mental > rest ∩ physical > rest ∩ control > rest): the superior parietal lobe, the posterior cerebellum, and the precentral gyrus. The cerebral ROIs were defined and masks for small-volume correction were created with probabilistic maps based on cytoarchitectonic data^92^. The cerebellar masks were based on the probabilistic atlas of the cerebellum provided by Diedrichsen et al.^88^. Significance was tested on the voxel level (*p* < 0.05, family-wise error [FWE]-corrected). We further calculated effect size maps using Cohen’s *d* for our main contrast of interest (physical > mental).

## Supplementary information

Supplementary Legends.

Supplementary Figure S1.

Supplementary Figure S2.

Supplementary Table S3.

Supplementary Table S4.

Supplementary Information.

## Data Availability

The data that support the findings of this study are available from the corresponding author upon request.
