# Supplementary material for: Practice modality of motor sequences impacts the neural signature of motor imagery
Source: Sci Rep. 2020 Nov 5;10:19176. doi: 10.1038/s41598-020-76214-y (PMC7645615; doi:10.1038/s41598-020-76214-y)
Supplement: Supplementary file 1 — Supplementary Legends. [file 41598_2020_76214_MOESM1_ESM.docx]

Practice modality of motor sequences impacts the neural signature of motor imagery

Britta Krüger^1^, Meike Hettwer^2^, Adam Zabicki^1^, Benjamin de Haas^4^,

Jörn Munzert^1^, and Karen Zentgraf*^3^

^1^Institute for Sports Science, Justus Liebig University Giessen, Germany

^2^Max Planck School of Cognition, Leipzig, Germany

^3^Institute of Sport Sciences, Goethe University Frankfurt, Germany

^4^Experimental Psychology, Justus Liebig University Giessen, Germany

Supplementary Figures

***Figure S1.*** *Additional behavioral data*. Subjects’ sequence execution sped up during practice, as indicated by **A)** the absolute duration and **B)** the constant error of sequence execution with respect to the presented model, pre and post practice interventions. During the scanner session, there was no systematic difference between imagery durations of sequences trained in different modalities **(C).**

***Figure S2.*** *Mental Chronometry.* Mental chronometry was assessed based on the duration of sequence execution after the intervention and imagery duration during the scanner session. **A)** Shows the overall relation between execution (ME) and imagery (MI) duration, split into **B)** mentally trained, **C)** physically trained and **D)** control sequences. *r_s_* = spearman’s rho, **p* < .05, ***p* < .01.
