# Supplementary figures and images for: Practice modality of motor sequences impacts the neural signature of motor imagery
Source: Sci Rep. 2020 Nov 5;10:19176. doi: 10.1038/s41598-020-76214-y (PMC7645615; doi:10.1038/s41598-020-76214-y)

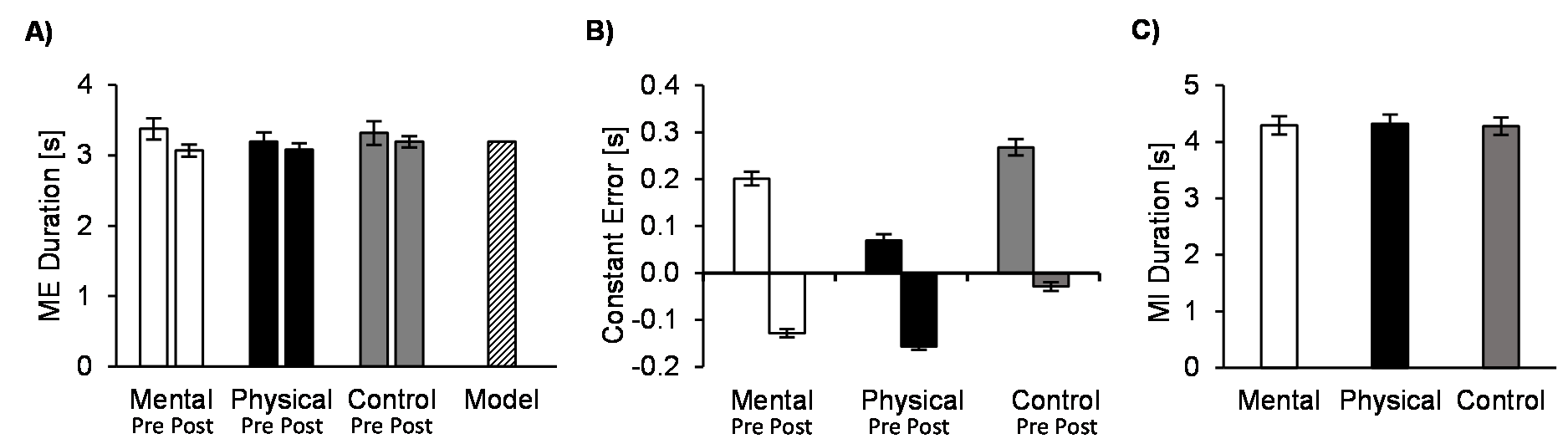

Supplement: Supplementary file 2 — Supplementary Figure S1. [file 41598_2020_76214_MOESM2_ESM.tif]

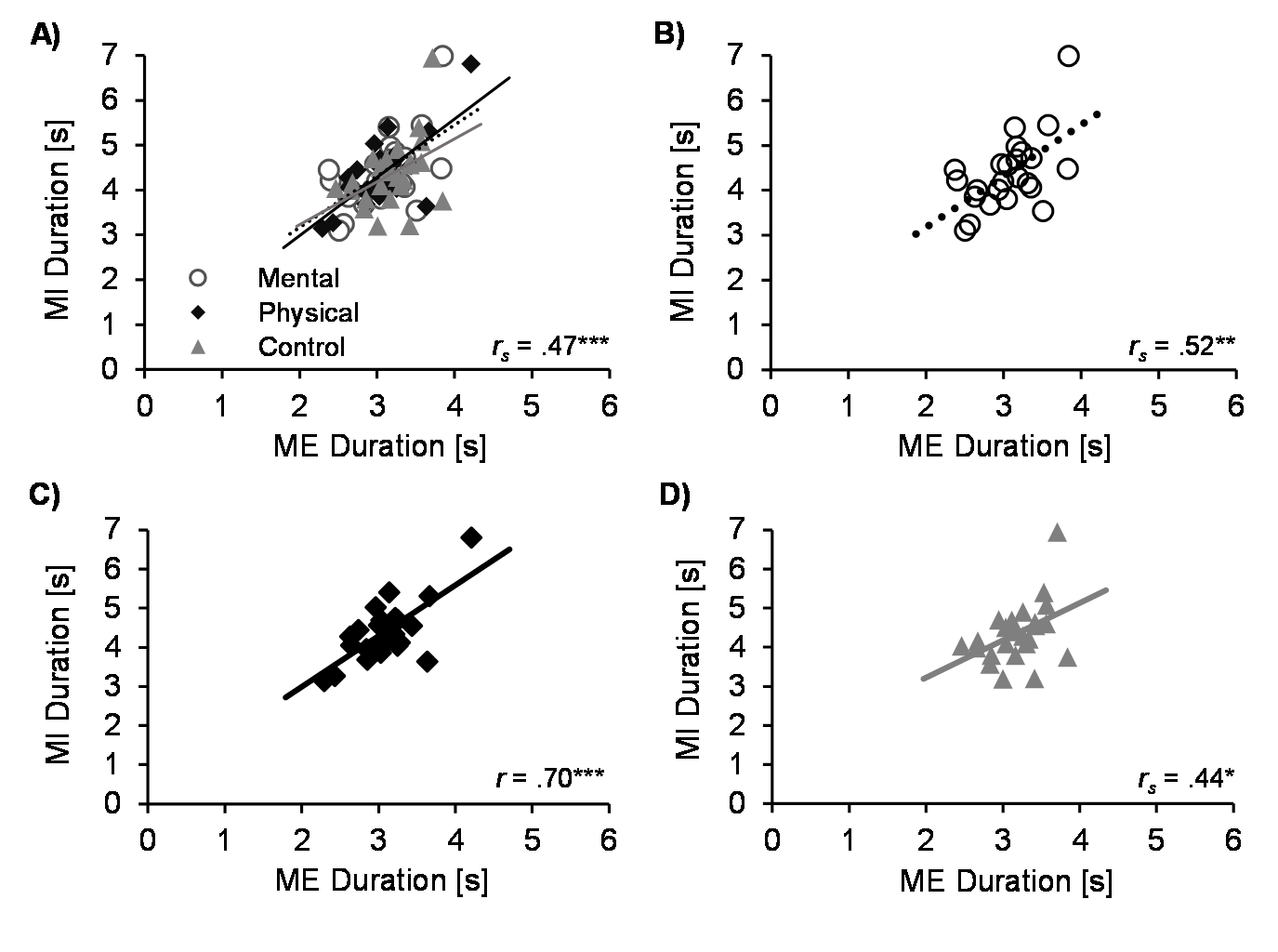

Supplement: Supplementary file 3 — Supplementary Figure S2. [file 41598_2020_76214_MOESM3_ESM.tif]
