# Supplementary material for: Practice modality of motor sequences impacts the neural signature of motor imagery
Source: Sci Rep. 2020 Nov 5;10:19176. doi: 10.1038/s41598-020-76214-y (PMC7645615; doi:10.1038/s41598-020-76214-y)
Supplement: Supplementary file 4 — Supplementary Table S3. [file 41598_2020_76214_MOESM4_ESM.docx]

Practice modality of motor sequences impacts the neural signature of motor imagery

Britta Krüger^1^, Meike Hettwer^2^, Adam Zabicki^1^, Benjamin de Haas^4^,

Jörn Munzert^1^, and Karen Zentgraf*^3^

^1^Institute for Sports Science, Justus Liebig University Giessen, Germany

^2^Max Planck School of Cognition, Leipzig, Germany

^3^Institute of Sport Sciences, Goethe University Frankfurt, Germany

^4^Experimental Psychology, Justus Liebig University Giessen, Germany

Table S3:

|  | Left/Right | | Coordinates of max. *t* value | | | *t* value |  | | |
| --- | --- | --- | --- | --- | --- | --- | --- | --- | --- |
| *Physical vs. Rest* | |  |  |  |  |  |  | | |
| Superior parietal gyrus (7A) | | L | -18 | -64 | 62 | 8.03 |  | | |
| Precentral gyrus | | L | -54 | -1 | 47 | 7.39 |  | | |
|  | |  |  |  |  |  |  | | |
| *Mental vs. Rest* | |  |  |  |  |  |  | | |
| Superior parietal gyrus (7A) | | L | -18 | -64 | 62 | 7.84 |  | | |
| Cuneus | | R | 18 | -97 | 8 | 6.75 |  | | |
|  | |  |  |  |  |  |  | | |
| *Control vs. Rest* | |  |  |  |  |  |  | | |
| Superior parietal gyrus (7A) | | L | -18 | -64 | 62 | 8.24 |  | | |
| Precentral gyrus | | L | -51 | -4 | 47 | 7.13 |  | | |
|  |  | |  |  |  |  |  | | |
| *Note.* MNI coordinates. Cluster size > 20, *p*<.05 (FWE-corrected). | | | | | | |  | | |
|  |  | |  | | |  | |  |  |
|  |  | |  |  |  |  | |  |  |
| *Physical vs. Mental* | | | |  |  |  | |  |  |
| Cerebellum VIIb | R | | 22 | -76 | -49 | 4.93 | |  |  |
| Cerebellum VIIIa | L | | -32 | -44 | -42 | 3.80 | |  |  |
|  |  | |  |  |  |  | |  |  |
| *Mental vs. Physical* | | | |  |  |  | |  |  |
| *No supra-threshold clusters* |  | |  |  |  |  | |  |  |
|  |  | |  |  |  |  | |  |  |
| *Physical vs. Control* | | | |  |  |  | |  |  |
| Precentral gyrus | | L | -42 | -10 | 44 | 5,41 | |  |  |
| Area hOc4v (V4) | | R | 42 | -73 | -16 | 5,27 | |  |  |
| Cuneus | | R | 12 | -85 | 41 | 5,14 | |  |  |
| Superior parietal cortex (7P) | | L | -3 | -85 | 38 | 4,63 | |  |  |
| Area hOc4la | | L | -51 | -79 | 8 | 4,90 | |  |  |
| Area TE 1.1 | | R | 36 | -28 | 8 | 4,88 | |  |  |
| Area hOc3v (V3) | | R | 30 | -94 | -13 | 4,68 | |  |  |
| Area hOc4lp | | R | 45 | -85 | -4 | 4,27 | |  |  |
| Area hOc4la | | R | 57 | -67 | 2 | 4,10 | |  |  |
| Precentral gyrus | | R | 24 | -22 | 74 | 4,61 | |  |  |
| Area TE3 | | L | -63 | -31 | 17 | 4,49 | |  |  |
| Area FG3 | | L | -33 | -61 | -10 | 4,34 | |  |  |
| Lingual gyrus | | L | -21 | -61 | -10 | 4,19 | |  |  |
| Precentral gyrus | | L | -24 | -22 | 71 | 4,32 | |  |  |
| Paracentral Lobe | | L | -15 | -19 | 77 | 4,01 | |  |  |
| Area 4a | | R | 9 | -31 | 56 | 4,06 | |  |  |
| Cerebellum VIIa | | R | 44 | -68 | -45 | 3,37 | |  |  |
|  | |  |  |  |  |  | |  |  |
| *Mental vs. Control* | |  |  |  |  |  | |  |  |
| Area FG3 | | L | -33 | -55 | -16 | 4,71 | |  |  |
| Area FG1 | | L | -33 | -76 | -7 | 4,44 | |  |  |
| Area hOc4la | | L | -36 | -79 | 2 | 3,90 | |  |  |
| Area hOc4v (V4) | | R | 39 | -76 | -10 | 4,51 | |  |  |
| Superior temporal gyrus | | R | -51 | -4 | -13 | 4,24 | |  |  |
| Putamen | | L | -27 | -10 | -1 | 3,94 | |  |  |
|  |  | |  |  |  |  | |  |  |
| *Note.* MNI coordinates. Cluster size > 20, *p* < .001 (uncorrected). | | | | | | | |  |  |
