# Supplementary material for: Practice modality of motor sequences impacts the neural signature of motor imagery
Source: Sci Rep. 2020 Nov 5;10:19176. doi: 10.1038/s41598-020-76214-y (PMC7645615; doi:10.1038/s41598-020-76214-y)
Supplement: Supplementary file 5 — Supplementary Table S4. [file 41598_2020_76214_MOESM5_ESM.docx]

**Table S4**. Additional subject characteristics.

Practice modality of motor sequences impacts the neural signature of motor imagery

Britta Krüger^1^, Meike Hettwer^2^, Adam Zabicki^1^, Benjamin de Haas^4^,

Jörn Munzert^1^, and Karen Zentgraf*^3^

^1^Institute for Sports Science, Justus Liebig University Giessen, Germany

^2^Max Planck School of Cognition, Leipzig, Germany

^3^Institute of Sport Sciences, Goethe University Frankfurt, Germany

^4^Experimental Psychology, Justus Liebig University Giessen, Germany

|  | **Mean (SD)** |
| --- | --- |
| **Cognitive tests** |  |
| KAI-N  ZVT  d2-R | 141.0 (20.9)  115.5 (9.40)  113.5 (12.2) |
| **VMIQ-2**  *Pre intervention* |  |
| External visual imagery  Internal visual imagery  Kinaesthetic imagery | 2.22 (0.73)  1.83 (0.53)  2.20 (0.68) |
| *Post intervention* |  |
| External visual imagery  Internal visual imagery  Kinaesthetic imagery | 2.07 (0.60)  1.70 (0.49)  2.13 (0.48) |

KAI-N: Lehrl & Blaha (2004); ZVT: Oswald & Roth (1987);
d2-R: Brickenkamp, Schmidt-Atzert & Liepmann (2010);
VMIQ-2: Roberts, Callow, Hardy, Markland & Bringer (2008)
