# Supplementary material for: Practice modality of motor sequences impacts the neural signature of motor imagery
Source: Sci Rep. 2020 Nov 5;10:19176. doi: 10.1038/s41598-020-76214-y (PMC7645615; doi:10.1038/s41598-020-76214-y)
Supplement: Supplementary file 6 — Supplementary Information. [file 41598_2020_76214_MOESM6_ESM.docx]

Practice modality of motor sequences impacts the neural signature of motor imagery

Britta Krüger^1^, Meike Hettwer^2^, Adam Zabicki^1^, Benjamin de Haas^4^,

Jörn Munzert^1^, and Karen Zentgraf*^3^

^1^Institute for Sports Science, Justus Liebig University Giessen, Germany

^2^Max Planck School of Cognition, Leipzig, Germany

^3^Institute of Sport Sciences, Goethe University Frankfurt, Germany

^4^Experimental Psychology, Justus Liebig University Giessen, Germany

**S5: Detailed information on the Training Sessions**

Each of the seven training sessions consisted of four blocks. In each block, the two mentally trained sequences as well as the two physically executed sequences were each presented once. The subjects then respectively executed or imagined the sequence five times in succession (corresponding to 20 trials per sequence). At the end of the intervention, participants had completed 280 imagery and 280 execution trials (i.e., 140 per sequence). In the physical condition, participants were asked to execute the observed sequence on a target grid - as presented in the stimulus material - positioned in front of them at eye level. For mentally trained sequences, participants closed their eyes and vividly imagined executing the shown sequence on the same target grid. The five trials following presentation (6 s) were separated by 3 s countdowns and were self-paced as participants indicated start and end points of a trial by pressing a button, providing a manipulation check based on mental chronometry (i.e., comparing durations of executed and imagined sequences). After every second training session they were asked to rate vividness of their imagery using a 7-point Likert-scale. For both modalities, they were instructed to reproduce the viewed sequence as accurately as possible, paying attention to spatiotemporal aspects. Participants were trained under inspection by the experimenter at all times.

In order to prepare the subjects for the scanner session, two additional actions were taken: Whereas for the first three blocks of a training session videos were used as cues (in order to specify spatiotemporal aspects, priming participants to show comparable behavior), static arrow images were implemented in block four. This should help participants get used to this type of stimulus which was later presented in the scanner. As participants worked on all six sequences during the scanner session – two of which were untrained – new sequences were implemented once every training session to prevent exceeding working memory engagement in the scanner. In addition, as during the scanner session participants worked on all six sequences – two of which were untrained – new sequences were implemented once every training session to prevent exceeding working memory engagement in the scanner.
